# Immune Imprinting and Implications for COVID-19

**DOI:** 10.3390/vaccines11040875

**Published:** 2023-04-20

**Authors:** Zhiqian Zhou, Julia Barrett, Xuan He

**Affiliations:** 1 Center for Virology and Vaccine Research, Beth Israel Deaconess Medical Center, Boston, MA 02115, USA; 2 Department of Respiratory and Critical Care Medicine, Frontiers Science Center for Disease-Related Molecular Network, West China Hospital, Sichuan University, Chengdu 610213, China; 3 Precision Medicine Research Center, West China Hospital, Sichuan University, Chengdu 610041, China

**Keywords:** B cells, immune imprinting, humoral immunity, vaccines, SARS-CoV-2, COVID-19

## Abstract

Immunological memory is the key source of protective immunity against pathogens. At the current stage of the COVID-19 pandemic, heterologous combinations of exposure to viral antigens during infection and/or vaccination shape a distinctive immunological memory. Immune imprinting, the downside of memory, might limit the generation of *de novo* immune response against variant infection or the response to the next-generation vaccines. Here, we review mechanistic basis of immune imprinting by focusing on B cell immunobiology and discuss the extent to which immune imprinting is harmful, as well as its effect on SARS-CoV-2 infection and vaccination.

## 1. Introduction

Immunological memory is crucial in the long-term protection against pathogens. Paradoxically, it can be both beneficial and harmful: the memory response primed by a viral strain leads to a certain level of pre-existing cross-reactive immunity. It may confer protection against severe outcomes caused by subsequent variant strain. However, under certain conditions, the memory B cells with high affinity and specificity which are induced by a primary viral infection can block the development of B cells in response to the subsequent infection of a novel but related virus, which is being referred to as immune imprinting.

COVID-19 (Coronavirus Disease 2019) caused by the infection of SARS-CoV-2 (Severe acute respiratory syndrome coronavirus 2) is a respiratory illness with symptoms ranging from mild to severe, and has had a profound impact on global health and the world economy since the beginning of its outbreak in late 2019 [1]. The virus has violently and rapidly spread worldwide, accumulating genetic mutations that give rise to new variants [2]. The emerging variants are expected as part of the viral evolution and are associated with changes in transmissibility and disease severity. So far, the vaccines most widely in use were developed based on the genetic sequence of the original Wuhan Hu-1 strain and target the spike protein on the surface of the coronavirus. As the spike protein is the pivot for triggering protective immune response, any changes in the spike protein can potentially compromise the effectiveness of SARS-CoV-2 vaccine. The variants of concern (VOC), such as Beta (B.1.351), Delta (B.1.617.2) and recently emerged Omicron with additional mutations on the spike, had all demonstrated immune escape of the vaccine-elicited immunity against SARS-CoV-2 [2,3,4,5,6,7,8,9]. In particular, the rising SARS-CoV-2 subvariants of Omicron BQ and XBB have rapidly expanded and posed a serious threat to vaccine efficacy, as well as have been observed to be largely resistant to clinically authorized therapeutic antibodies [10,11]. Confronting the great challenge by emerging VOCs, the Omicron-adapted bivalent vaccines have been developed in record time, and proved to be effective at neutralizing Omicron subvariants, especially for BA.2.75.2, BQ.1.1, and XBB subvariants [11,12]. Memory B cells are a powerful defense against emerging VOCs as they provide long-lived “antibody memory” with the capacity to adapt to the diversification of viral antigens [13,14,15]. However, pre-existing and cross-reactive memory B cell pools may complicate the vaccine-induced memory B cell response by immune imprinting. Given the distinctive histories of SARS-CoV-2 infection and vaccination, different immune repertoires can be primed at population level, which can drive difference of immune response to subsequent variant infection or the variant-adapted vaccine booster [16]. Understanding the impact of immune imprinting on the response to SARS-CoV-2 infection and vaccination is crucial for improving strategies to better combat the emerging VOCs in the COVID-19 pandemic.

## 2. B Cell Memory Induced by SARS-CoV-2

**Figure 1:**
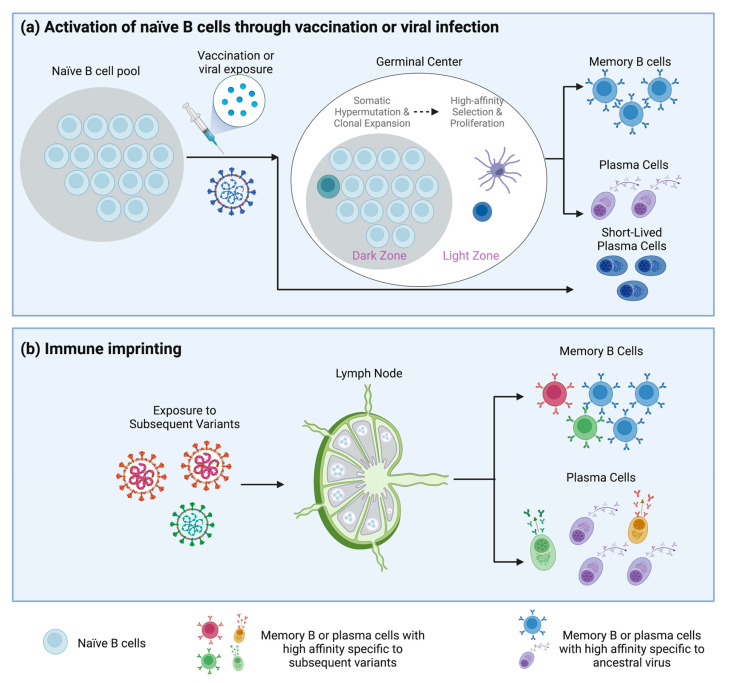
Establishment of B cell memory and immune imprinting. (**a**) Activation of memory B cells through vaccination or viral infection. Upon exposure to viral antigens by vaccination or infection, a germinal center starts to form. In the dark zone of the germinal center, optimal B cells with high-affinity BCR expressed on the surface can be selected for clonal expansion with the help of T_FH_ cells and differentiate into long-term memory B cells and plasma cells. Some B cells can rapidly differentiate into short-lived plasma cells with low-binding affinity that are formed in the extrafollicular sites of secondary lymphoid organs. (**b**) The impact of immune imprinting on humoral immunity. In the presence of subsequent viral variants, the lymph nodes that have previously generated memory cells after exposure to ancestral viral antigens tend to produce a relatively higher number of memory B cells expressing antibodies against the ancestral virus compared to the B cells targeting the viral variants. (Created with BioRender.com).

Upon exposure to viral antigen during infection, naïve B cells activate through the B cell receptor (BCR) and migrate to the B cell follicle in secondary lymphoid tissues [14,17]. A germinal center forms as the activated B cell moves to the B cell follicle to present its antigen to cognate T follicular helper (T_FH_) cells, which provide CD40 and CD40 ligand (CD40L) signaling to promote survival [14,18,19,20]. CD40L and T_FH_ cell-derived cytokines enable the B cell to undergo clonal expansion and somatic hypermutation (SHM) within the dark zone of the forming germinal center [14,20]. During this period, the germinal center B cell proliferates rapidly and replicate every 4 to 6 h to allow for extensive antigen specific evolution [21,22]. A subset of follicular B cells undergoes immunoglobulin class-switch recombination to express IgG and differentiates into short-lived plasmablasts that provide low-antigen affinity antibodies during the early adaptive immune response and contribute towards the B cell pool antigen affinity diversity [14,19,23]. The B cells that acquire high-affinity mutations for the antigen enter the germinal center light zone where they compete with other high-affinity B cells for antigen presentation to T cells [14,19]. Successful competition in this process allows for the generation of high affinity memory B cells and plasma cells, both of which are sources of antigen-specific antibodies, one of the most important weapons against infectious disease [14,16,19] (Figure 1a). The lifespan of these long-lived plasma cells and the durability of the humoral response against pathogens is determined by the magnitude and intensity of B cell signaling during the generation of antigen-stimulated immune response in germinal center [14,23,24]. 

After terminal differentiation in the germinal center, newly formed plasma cells with high antigen affinity migrate to the bone marrow where, in the absence of antigens, they survive and secrete high-affinity antibodies [14,24] which provide sustained protection upon reinfection or breakthrough infection after vaccination [16,17,20,25]. During SARS-CoV-2 infection, these long-lived plasma cells can secrete neutralizing antibodies (NAbs) targeting SARS-CoV-2 spike for up to 240 days post antigen exposure, despite decreasing antibody levels following infection [25,26]. Furthermore, serum collected in convalescent patients demonstrated that the declining antibody titers were not linear but followed a biphasic pattern, wherein the decline was faster during the first 1 to 4 months after infection, and slower during the subsequent 5 to 16 months [26,27]. This accelerated early reduction in circulating antibodies against spike followed by a more gradual decline can be attributed to the humoral immune conversion of antibodies secreted by short-lived plasma cells into those generated by long-lived plasma cells residing in the bone marrow [26]. Distinct kinetics of immunoglobulin isotypes were also observed with a relatively faster decline of IgM and IgG antibodies targeting the receptor binding domain (RBD) of SARS-CoV-2 spike proteins while anti-RBD IgA levels remain more consistent at 1.3 to 6.2 months post-infection [28]. In the non-human primate model, spike-specific plasma cells persisted beyond 300 days following SARS-CoV-2 vaccination, and were highly correlated with the magnitude of humoral responses in the long-term follow-up [29]. Innate immunity, such as type I IFN response, might have an impact on the early programming of B cell differentiation and subsequently the quality and quantity of plasma cells following exposure to SARS-CoV-2 antigen [29,30,31].

Long-lived memory B cells provide a quiescent and durable source of high-affinity antibodies against pathogens [26]. After re-exposure to antigens, memory B cells rapidly proliferate and differentiate into antibody-secreting cells, eliciting a rapid and strong secondary humoral immune response [26,32]. During SARS-CoV-2 infection, memory B cells are generated with specificity towards their cognate antigen, including the broadly neutralizing antigen epitopes targeting SARS-CoV-2 spike through affinity maturation [25,33]. In the analysis of individuals with asymptomatic and symptomatic SARS-CoV-2 infection, spike-binding memory B cells were identifiable in 77% of participants in both symptomatic and asymptomatic participants with no significant difference in frequency between the two groups [34]. However, memory B cells specific to SARS-CoV-2 were sparse during infection with 0.008–0.1% of B cells predominantly expressing IgM or IgG1 in COVID-19 patients [25]. The poly-reactive memory B cell pool allowed for a more flexible and cross-reactive response in antigen binding [16], which might be particularly important in fighting against the highly contagious VOCs in the current COVID-19 pandemic. With no exposure to variant antigens, 10% of memory B cells can target the variant epitope better than the ancestral viral antigen, demonstrating the potential breadth of protection mediated by memory B cells [16,35]. In SARS-CoV-2 infection, the neutralizing antibodies against spike protein mainly display a lack of SHM, suggesting SARS-CoV-2 is relatively untroubled to be targeted and neutralized as compared to other virus such as HIV, for which the potent NAbs are not readily to be induced by infection or vaccination [30,36]. Although the analyses in convalescent individuals revealed the memory B cells against RBD epitopes were expanded but largely producing antibodies with modest neutralizing activity [30,37], these less matured memory B cells can re-enter the germinal center to undergo further rounds of SHM after exposure to viral variants [22].

The emerging VOCs, such as the Omicron sublineages, have demonstrated dramatically increased ability to evade neutralizing antibodies, even those from people who received the bivalent mRNA booster vaccine or who are immunized and had breakthrough infection [38,39,40]. Given the alarming reduction in neutralizing antibodies against immune-escaped VOCs, the durability and flexibility of memory B cells has proved highly important in the coevolution of variants: the circulating or tissue-resident memory B cells can maintain antigen specificity as well as being capable of quickly adapting to new variants, thus being highly involved in countering the rapidly evolving SARS-CoV-2 [41,42]. Memory B cell repertoires from convalescent COVID-19 patients revealed that SARS-CoV-2 specific memory B cells can persist in lymphoid and mucosal tissues for up to 6 months post-infection [22,43,44,45]. In addition, with decreased neutralizing antibody titers following SARS-CoV-2 infection over time, memory B cells against RBD not only preserved well for up to 8 months in circulation [34], but also continue to evolve in convalescence with advancing somatic mutations [25,28,30,33]. At six months post-infection, the memory B cell compartment underwent clonal turnover with new and expanded clones expressing antibodies of increased potency and better resistance to RBD mutations, which might be driven by the SARS-CoV-2 antigens remaining in the small intestinal epithelium [28]. Of note, a recent study demonstrated that a third antigenic exposure by Delta breakthrough infection was capable of eliciting Delta-specific memory B response and also expanded the breadth and potency of memory B pool, while a fourth antigenic exposure by Omicron breakthrough did not boost the overall memory B cell response and showed little effect on the breadth and potency of memory B cells [41]. The limitations in boosting potent memory B cell response present a further challenge in the development of strain-specific vaccines to fight against future VOCs.

## 3. Immune Imprinting in COVID-19 Pandemic

### 3.1. Immune Imprinting

As mentioned earlier, the general theory of immunology describes the rule of clonal selection of the best matching B cells in the germinal center. However, many studies have reported a controversial phenomenon that when a second antigen involves in the existing humoral response, the boosted humoral immunity elicited by the second antigen not only cross-reacts but also reacts better with the first antigen presented to the immune system [46,47,48]. This phenomenon is called immune imprinting, also known as ‘original antigenic sin’. In viral infection, immune imprinting is manifesting as the secondary immune response to successive viral variants can be shaped by the ancestral virus exposed to the immune system and is skewed towards the ancestral immunogen.

Immune imprinting in cross-reactive humoral immunity between two related viruses with antigenic similarities has been elegantly explored and described by many groups who are focusing on emerging widespread and pathogenic viruses, such as influenza virus and dengue virus. In early studies on influenza, it was found that the humoral immunologic memory generated from previous influenza viral exposure can influence the development of cross-reactive NAbs to the subsequent influenza viral strains or vaccinations [48]. The study on the historical serum samples from individuals revealed that the antibody subtype that cross-reacts to antecedent variants of influenza virus, consistently has the highest titers. These data suggest the phenomenon of back-boosting with the original antibodies by subsequent infection [49]. Accordingly, analyses on B cell repertories also showed that memory B cells generated after exposure to sequential infections with more advanced influenza viral variants or variant-adjusted vaccinations can still have cross-reactivity to antecedent influenza viral strains experienced earlier in life [13,50,51]. Of note, the analyses of monoclonal antibodies produced by B cells following influenza vaccination demonstrated that individuals with a low level of pre-existing antibodies were more likely to develop antibodies against more conserved hemagglutinin (HA) stalk region. While those with a higher level of pre-existing antibodies mainly generated antibodies targeting the HA head, suggesting that pre-existing head antibodies blocked the development of protective HA stalk-specific antibodies, a phenomenon showing negative modulation by imprinting effect [52]. Immune imprinting also occurs in dengue viral infection as suggested by the boosting of cross-reactive antibody responses upon re-exposure to a different serotype but with a higher level of antibodies specific to the original virus compared to those against the secondary viral strain [53,54].

Although many studies have reported consistent observations of immune imprinting in viral infection, the precise mechanism of immune imprinting remains obscure. One supposition is that within the germinal center niches, those matured B cells induced by prior infection and exhibiting high-affinity BCR can outstrip naïve B cells that need higher signaling thresholds for activation, and rapidly differentiate into antibody-secreting plasma cells following re-exposure to their cognate antigen. Thus, when encountering a related but antigenically distant pathogens, pre-existing memory B cell response can not only be worthless but also compromise the ability of naïve B cells in response to new antigens (Figure 1b). Furthermore, under survival pressure, the virus continues evolving by mutation of antigenic epitopes, often the most exposed epitopes expressed on the surface, to escape from the adaptive immune system [55,56]. However, some conserved and potentially non-neutralizing epitopes of the virus remain unchanged. Those conserved epitopes being repeatedly exposed could become the dominant target for memory B cell activation, thus interfering with the B cells responding to key neutralizing epitopes of new pathogen.

### 3.2. Immunological Imprinting in SARS-CoV-2 Infection

SARS-CoV-2, as a positive-stranded RNA virus, is prone to genetic evolution with a persistent generation of mutations to adapt to its hosts, which leads to the emergence of multiple divergent variants. The VOC Omicron B.1.1.529 was first reported to WHO in November 2021. Omicron and its subvariants with the fast transmission have displaced the prior VOCs, and become the most prevalent circulating SARS-CoV-2 variants nowadays, leading to a large population of breakthrough infections [8,9]. The Omicron variants were analyzed and found to have more than 30 mutations in spike region, with 15 mutations corresponding to the epitopes on RBD. These mutations synergize and create better opportunities for the viruses to escape immune surveillance, while some conserved RBD epitopes remain unmutated [40]. These conserved epitopes share cross-reactivity with the prior strains, including the ancestral Wuhan Hu-1 strain. This explains why even after multiple rounds of drastic antigen drifts in SARS-CoV-2 since the wildtype Wuhan Hu-1, the booster vaccine with the Wuhan Hu-1 strain remain effective in preventing severe conditions among general populations under the latest waves of dominant infections caused by the latest VOCs such as Omicron subvariants [57]. Nevertheless, in a longitudinal study, humoral immunity against VOCs were significantly compromised in the triple-vaccinated cohort due to immune imprinting primed by prior exposure to the ancestral Wuhan Hu-1 strain, while COVID-19 patients associated with VOC infections displayed serological profiles specific to variant epitopes [58]. Accordingly, a group analyzed fully vaccinated and boosted subjects with distinct SARS-CoV-2 infection histories to explore the cross-protectivity against Omicron, and observed “hybrid immune damping”, a phenomenon which was attributable to the immune imprinting effect that instead of targeting the new variant, NAbs specific to Wuhan Hu-1 wildtype was back-boosted after Omicron infection [39]. In addition to SARS-CoV-2 variants, immune imprinting by prior seasonal coronavirus infections can also potentially modulate the humoral immunity against SARS-CoV-2 infection. In hospitalized COVID-19 patients, there was a strong boosting of spike protein epitopes of other seasonal coronaviruses that target conserved epitopes of OC43 and HKU1 betacoronaviruses in the longitudinal immune profile. This seemingly has a negative impact on the efficacy of COVID-19 vaccine by hindering the induction of *de novo* NAbs against SARS-CoV-2 [59]. These data suggest that the immune imprinting is not only present in the influenza but also in the COVID-19 pandemic. The distinct antibody and B cell profiles in hybrid immunity with different combinations of SARS-CoV-2 vaccination and infection, which are highly involved in immune imprinting, need to be systemically investigated for further COVID-19 booster strategy.

### 3.3. Impact of Immune Imprinting on COVID-19 Vaccine

The approved or authorized COVID-19 vaccines are developed by different platforms including mRNA [60,61], viral vectors that express full-length spike proteins [62,63,64], inactivated whole virus [65,66,67] or purified spike proteins [68,69]. All of these vaccines are designed based on the ancestral SARS-CoV-2 genomic sequence, Wuhan Hu-1, which was released in 2020 [70]. These COVID-19 vaccines are highly effective against the predominant circulating virus that were genetically close to the ancestral strain, such as Alpha (B.1.1.7) variant. However, fast-spreading SARS-CoV-2 variants with multiple mutations all through their genome have emerged in different countries all over the world. Among the VOCs, those with mutations critically in neutralizing antibody epitope RBD affect the biological function of SARS-CoV-2 and are capable of escaping vaccine-induced immune response, therefore posting a great challenge for vaccine effectiveness. Updating first-generation vaccines by changing the original spike into the spike from the new dominant variant has been proposed by vaccine manufacturers. Recurrent vaccination with either prototype vaccine or variant-adapted vaccine will become common. Immune imprinting might negatively impact the effectiveness of vaccine development for evolving variants: the primary negative effect is its intervention with naïve B cells in generating more specific antibodies with high affinity to the current circulating VOC; secondly, the repeated exposure to conserved but non-neutralizing viral epitopes might result in the dominant humoral immunity with non-functional antibodies.

Early studies have revealed a certain level of immune imprinting during immunization with SARS-CoV-2 variant-targeting vaccines following the original vaccine series. Back-boosting of memory B cells responding to the ancestral viral strain was observed in monkeys vaccinated with Beta variant-targeting vaccine following immunization with prototype vaccine [29]. Consistent with the preclinical data from animal model, in a human clinical trial, boosting with Beta variant-adapted mRNA vaccine after two-shot prototype mRNA vaccine led to higher NAb titers specific to the original strain than that against Beta variant, although variant-adapted mRNA vaccine can still develop a rapid anamnestic response targeting key VOCs, and appeared to generate a numerically higher level of NAbs against Beta variant than prototype mRNA vaccine [71]. Therefore, the question is raised as to whether there’s extra benefit from boosting with the variant-adapted vaccine when the recall of B cell and antibody response elicited by homologous boosting with the original vaccine might have already been sufficient to offer protection against infection and server disease caused by circulating variant. Cromer’s group attempted to predict the efficacy of variant-modified vaccine boosters based on the meta-analysis of NAb data from clinical studies that compare the booster dose with the original vaccine or variant-adapted vaccines. They found that individuals with an updated variant vaccine showed in average 1.5-fold higher antibody levels than those with the original vaccine. Interestingly, the variant vaccine still shows benefits even when the vaccine does not entirely match the viral strain [72].

Under the pressure of the unprecedented growth rate of the VOC Omicron which has dominated globally, Omicron-adapted vaccines were developed and tested in record time by different groups. A report from NIAID and Moderna (and others) based on a study in non-human primates suggested that following primary vaccination with standard Moderna mRNA, monkeys boosted with either the Omicron-adjusted mRNA vaccine or the homologous mRNA vaccine elicited comparable humoral response against Omicron variant [12]. Both vaccine booster regimens enhanced neutralization of Omicron, and offered protection in the lung after viral challenge. Immune imprinting appeared to highly involve in driving the similarly high frequencies of memory B cells as measured in non-human primates boosted with the original or Omicron-adapted vaccine. It is plausible that imprinted memory B cells induced by the original mRNA vaccine dominate the response to the booster vaccine. Thus, based on the small-scale preclinical study, at least in the short term, boosting with Omicron-mRNA vaccine has not yet presented big advantage over the original mRNA vaccine regarding the induction of protective NAbs against variant as well as control of viral replication after challenge, and immune imprinting seemingly involved in damping the B cell response to variant epitopes. The Omicron-matched vaccine with accepted safety and immunogenicity profile is further tested in a clinical trial at large cohort scale. The bivalent SARS-CoV-2 mRNA vaccine, which contains two spike components of ancestral SARS-CoV-2 and Omicron variant BA.1 lineage, was administrated as a second booster in individuals who had previously received two primary series and the first booster dose of the original mRNA vaccine [73]. The group that received bivalent vaccine generated a 1.6-fold higher NAbs against Omicron BA.1 variant, and 1.4-fold higher NAbs against BA.4/5 variant as compared to those boosted with the original vaccine. Thus, according to the data from human clinical trial, the bivalent Omicron-adapted mRNA vaccine, seemingly also being affected by immune imprinting, is only modestly superior to the prototype mRNA vaccine for induction of NAb response against the current circulating Omicron variant. On 31 August 2022, the U.S. Food and Drug Administration (FDA) has authorized the bivalent formulations of mRNA vaccines (half for ancestral strain, half for Omicron BA.4/5) from Moderna and Pfizer for the use as a single booster shot, and approximately 12% of U.S. population had received the bivalent mRNA booster. Of note, recent data from a pair of studies on subjects with bivalent mRNA booster suggested that bivalent mRNA vaccine did not elicit superior NAb response against BA.4/5 as compared with the monovalent vaccine by pseudovirus assay, which is probably attributable to the immune imprinting by previous SARS-CoV-2 antigen exposure [74,75]. Although the bivalent vaccine has become a new tool in response to the emerging VOC Omicron, great challenge may be posed by immune imprinting which inhibits the development of memory B cells and NAb against new epitopes of Omicron. The efficacy of the bivalent COVID-19 vaccine in preventing viral replication and transmission, especially in people with distinct histories of viral antigen exposure or in other words, with disparate immune imprinting, needs to be monitored closely over time.

## 4. Conclusions

Immunological memory is a double-edged sword: it can offer protection against novel but closely related pathogens or block the development of NAbs against related but antigenically distant pathogens. Due to the constant evolution of SARS-CoV-2, recurrent vaccination and breakthrough infection have been common, making the population-level immunity against COVID-19 diverse and complicated. How the immune imprinting influences the SARS-CoV-2 infection or vaccination is yet to be comprehensively determined and should be considered when evaluating the efficacy of the updated vaccines at a population level. To combat the emerging SARS-CoV-2 variants that favor immune evasion, reformulating COVID-19 vaccine matched to the dominant variant is being pursued. Further COVID-19 vaccine strategies might need to be developed for overcoming the negative modulation by imprinted immune response. Boosting immunity with well-designed immunodominant RBD proteins of variants or spacing out vaccine shots over a longer time interval could potentially skew the humoral immunity toward key RBD epitopes of emerging VOCs.

## Data Availability

Not applicable.

## References

[1] Fauci A.S., Lane H.C., Redfield R.R. (2020). COVID-19—Navigating the Uncharted. N. Engl. J. Med..

[2] Tregoning J.S., Flight K.E., Higham S.L., Wang Z., Pierce B.F. (2021). Progress of the COVID-19 Vaccine Effort: Viruses, Vaccines and Variants versus Efficacy, Effectiveness and Escape. Nat. Rev. Immunol..

[3] Kurhade C., Zou J., Xia H., Liu M., Chang H.C., Ren P., Xie X., Shi P. (2022). Low Neutralization of SARS-CoV-2 Omicron BA.2.75.2, BQ.1.1, and XBB.1 by Parental MRNA Vaccine or a BA.5-Bivalent Booster. Nat. Med..

[4] Lopez Bernal J., Andrews N., Gower C., Gallagher E., Simmons R., Thelwall S., Stowe J., Tessier E., Groves N., Dabrera G. (2021). Effectiveness of COVID-19 Vaccines against the B.1.617.2 (Delta) Variant. N. Engl. J. Med..

[5] Andrews N., Stowe J., Kirsebom F., Toffa S., Rickeard T., Gallagher E., Gower C., Kall M., Groves N., O’Connell A.-M. (2022). COVID-19 Vaccine Effectiveness against the Omicron (B.1.1.529) Variant. N. Engl. J. Med..

[6] Planas D., Veyer D., Baidaliuk A., Staropoli I., Guivel-Benhassine F., Rajah M.M., Planchais C., Porrot F., Robillard N., Puech J. (2021). Reduced Sensitivity of SARS-CoV-2 Variant Delta to Antibody Neutralization. Nature.

[7] Shinde V., Bhikha S., Hoosain Z., Archary M., Bhorat Q., Fairlie L., Lalloo U., Masilela M.S.L., Moodley D., Hanley S. (2021). Efficacy of NVX-CoV2373 COVID-19 Vaccine against the B. 1.351 Variant. N. Engl. J. Med..

[8] Callaway E. (2021). Heavily Mutated Omicron Variant Puts Scientists on Alert. Nature.

[9] Callaway E., Ledford H. (2021). How Bad Is Omicron? What Scientists Know so Far. Nature.

[10] Cao Y., Jian F., Wang J., Yu Y., Song W., Yisimayi A., Wang J., An R., Chen X., Zhang N. (2023). Imprinted SARS-CoV-2 Humoral Immunity Induces Convergent Omicron RBD Evolution. Nature.

[11] Davis-Gardner M.E., Lai L., Wali B., Samaha H., Solis D., Lee M., Porter-Morrison A., Hentenaar I.T., Yamamoto F., Godbole S. (2022). Neutralization against BA.2.75.2, BQ.1.1, and XBB from MRNA Bivalent Booster. N. Engl. J. Med..

[12] Gagne M., Moliva J.I., Foulds K.E., Andrew S.F., Flynn B.J., Werner A.P., Wagner D.A., Teng I.-T., Lin B.C., Moore C. (2022). MRNA-1273 or MRNA-Omicron Boost in Vaccinated Macaques Elicits Similar B Cell Expansion, Neutralizing Responses, and Protection from Omicron. Cell.

[13] Wrammert J., Koutsonanos D., Li G.-M., Edupuganti S., Sui J., Morrissey M., McCausland M., Skountzou I., Hornig M., Lipkin W.I. (2011). Broadly Cross-Reactive Antibodies Dominate the Human B Cell Response against 2009 Pandemic H1N1 Influenza Virus Infection. J. Exp. Med..

[14] Laidlaw B.J., Ellebedy A.H. (2022). The Germinal Centre B Cell Response to SARS-CoV-2. Nat. Rev. Immunol..

[15] Roy S., Williams C.M., Wijesundara D.K., Furuya Y. (2020). Impact of Pre-Existing Immunity to Influenza on Live-Attenuated Influenza Vaccine (LAIV) Immunogenicity. Vaccines.

[16] Palm A.-K.E., Henry C. (2019). Remembrance of Things Past: Long-Term B Cell Memory after Infection and Vaccination. Front. Immunol..

[17] Treanor B. (2012). B-cell Receptor: From Resting State to Activate. Immunology.

[18] Huang C. (2020). Germinal Center Reaction. B Cells Immun. Toler..

[19] Tomohiro K., Wataru I. (2015). Memory B Cells. Nat. Rev. Immunol..

[20] Nutt S.L., Hodgkin P.D., Tarlinton D.M., Corcoran L.M. (2015). The Generation of Antibody-Secreting Plasma Cells. Nat. Rev. Immunol..

[21] Lee J.H., Sutton H.J., Cottrell C.A., Phung I., Ozorowski G., Sewall L.M., Nedellec R., Nakao C., Silva M., Richey S.T. (2022). Long-Primed Germinal Centres with Enduring Affinity Maturation and Clonal Migration. Nature.

[22] Gitlin A.D., Shulman Z., Nussenzweig M.C. (2014). Clonal Selection in the Germinal Centre by Regulated Proliferation and Hypermutation. Nature.

[23] Viant C., Weymar G.H.J., Escolano A., Chen S., Hartweger H., Cipolla M., Gazumyan A., Nussenzweig M.C. (2020). Antibody Affinity Shapes the Choice between Memory and Germinal Center B Cell Fates. Cell.

[24] Amanna I.J., Slifka M.K. (2010). Mechanisms That Determine Plasma Cell Lifespan and the Duration of Humoral Immunity. Immunol. Rev..

[25] Hartley G.E., Edwards E.S.J., Aui P.M., Varese N., Stojanovic S., McMahon J., Peleg A.Y., Boo I., Drummer H.E., Hogarth P.M. (2020). Rapid Generation of Durable B Cell Memory to SARS-CoV-2 Spike and Nucleocapsid Proteins in COVID-19 and Convalescence. Sci. Immunol..

[26] Turner J.S., Kim W., Kalaidina E., Goss C.W., Rauseo A.M., Schmitz A.J., Hansen L., Haile A., Klebert M.K., Pusic I. (2021). SARS-CoV-2 Infection Induces Long-Lived Bone Marrow Plasma Cells in Humans. Nature.

[27] Yang Y., Yang M., Peng Y., Liang Y., Wei J., Xing L., Guo L., Li X., Li J., Wang J. (2022). Longitudinal Analysis of Antibody Dynamics in COVID-19 Convalescents Reveals Neutralizing Responses up to 16 Months after Infection. Nat. Microbiol..

[28] Gaebler C., Wang Z., Lorenzi J.C.C., Muecksch F., Finkin S., Tokuyama M., Cho A., Jankovic M., Schaefer-Babajew D., Oliveira T.Y. (2021). Evolution of Antibody Immunity to SARS-CoV-2. Nature.

[29] He X., Aid M., Chandrashekar A., Yu J., McMahan K., Wegmann F., Jacob-Dolan C., Maron J.S., Atyeo C., Wan H. (2022). A Homologous or Variant Booster Vaccine after Ad26. COV2. S Immunization Enhances SARS-CoV-2–Specific Immune Responses in Rhesus Macaques. Sci. Transl. Med..

[30] Sette A., Crotty S. (2021). Adaptive Immunity to SARS-CoV-2 and COVID-19. Cell.

[31] Kasturi S.P., Skountzou I., Albrecht R.A., Koutsonanos D., Hua T., Nakaya H.I., Ravindran R., Stewart S., Alam M., Kwissa M. (2011). Programming the Magnitude and Persistence of Antibody Responses with Innate Immunity. Nature.

[32] Wherry E.J., Barouch D.H. (2022). T Cell Immunity to COVID-19 Vaccines. Science.

[33] Mahrokhian S.H., Tostanoski L.H., Jacob-Dolan C., Zahn R.C., Wegmann F., McMahan K., Yu J., Gebre M.S., Bondzie E.A., Wan H. (2022). Durability and Expansion of Neutralizing Antibody Breadth Following Ad26. COV2. S Vaccination of Mice. NPJ Vaccines.

[34] Dan J.M., Mateus J., Kato Y., Hastie K.M., Yu E.D., Faliti C.E., Grifoni A., Ramirez S.I., Haupt S., Frazier A. (2021). Immunological 432 Memory to SARS-CoV-2 Assessed for up to 8 Months after Infection. Science.

[35] Purtha W.E., Tedder T.F., Johnson S., Bhattacharya D., Diamond M.S. (2011). Memory B Cells, but Not Long-Lived Plasma Cells, Possess Antigen Specificities for Viral Escape Mutants. J. Exp. Med..

[36] Haynes B.F. (2021). SARS-CoV-2 and HIV-1—A Tale of Two Vaccines. Nat. Rev. Immunol..

[37] Robbiani D.F., Gaebler C., Muecksch F., Lorenzi J.C.C., Wang Z., Cho A., Agudelo M., Barnes C.O., Gazumyan A., Finkin S. (2020). Convergent Antibody Responses to SARS-CoV-2 in Convalescent Individuals. Nature.

[38] Cao Y., Su B., Guo X., Sun W., Deng Y., Bao L., Zhu Q., Zhang X., Zheng Y., Geng C. (2020). Potent Neutralizing Antibodies against SARS-CoV-2 Identified by High-Throughput Single-Cell Sequencing of Convalescent Patients’ B Cells. Cell.

[39] Wang K., Jia Z., Bao L., Wang L., Cao L., Chi H., Hu Y., Li Q., Zhou Y., Jiang Y. (2022). Memory B Cell Repertoire from Triple Vaccinees against Diverse SARS-CoV-2 Variants. Nature.

[40] He B., Liu S., Wang Y., Xu M., Cai W., Liu J., Bai W., Ye S., Ma Y., Hu H. (2021). Rapid Isolation and Immune Profiling of SARS-CoV-2 Specific Memory B Cell in Convalescent COVID-19 Patients via LIBRA-Seq. Signal Transduct. Target. Ther..

[41] Wang Z., Zhou P., Muecksch F., Cho A., Ben Tanfous T., Canis M., Witte L., Johnson B., Raspe R., Schmidt F. (2022). Memory B Cell Responses to Omicron Subvariants after SARS-CoV-2 MRNA Breakthrough Infection in Humans. J. Exp. Med..

[42] Hachmann N.P., Miller J., Collier A.Y., Ventura J.D., Yu J., Rowe M., Bondzie E.A., Powers O., Surve N., Hall K. (2022). Neutralization Escape by SARS-CoV-2 Omicron Subvariants BA.2.12.1, BA.4, and BA.5. N. Engl. J. Med..

[43] Park Y.-J., Pinto D., Walls A.C., Liu Z., De Marco A., Benigni F., Zatta F., Silacci-Fregni C., Bassi J., Sprouse K.R. (2022). Imprinted Antibody Responses against SARS-CoV-2 Omicron Sublineages. Science.

[44] Reynolds C.J., Pade C., Gibbons J.M., Otter A.D., Lin K.-M., Muñoz Sandoval D., Pieper F.P., Butler D.K., Liu S., Joy G. (2022). Immune Boosting by B.1.1.529 (Omicron) Depends on Previous SARS-CoV-2 Exposure. Science.

[45] Cao Y., Wang J., Jian F., Xiao T., Song W., Yisimayi A., Huang W., Li Q., Wang P., An R. (2022). Omicron Escapes the Majority of Existing SARS-CoV-2 Neutralizing Antibodies. Nature.

[46] Francis T. (1960). On the Doctrine of Original Antigenic Sin. Proc. Am. Philos. Soc..

[47] Ndifon W. (2015). A Simple Mechanistic Explanation for Original Antigenic Sin and Its Alleviation by Adjuvants. J. R. Soc. Interface.

[48] Henry C., Palm A.-K.E., Krammer F., Wilson P.C. (2018). From Original Antigenic Sin to the Universal Influenza Virus Vaccine. Trends Immunol..

[49] Zhang A., Stacey H.D., Mullarkey C.E., Miller M.S. (2019). Original Antigenic Sin: How First Exposure Shapes Lifelong Anti–Influenza Virus Immune Responses. J. Immunol..

[50] Cortina-Ceballos B., Godoy-Lozano E.E., Téllez-Sosa J., Ovilla-Muñoz M., Sámano-Sánchez H., Aguilar-Salgado A., Gómez-Barreto R.E., Valdovinos-Torres H., López-Martínez I., Aparicio-Antonio R. (2015). Longitudinal Analysis of the Peripheral B Cell Repertoire Reveals Unique Effects of Immunization with a New Influenza Virus Strain. Genome Med..

[51] Tan Y.-C., Blum L.K., Kongpachith S., Ju C.-H., Cai X., Lindstrom T.M., Sokolove J., Robinson W.H. (2014). High-Throughput Sequencing of Natively Paired Antibody Chains Provides Evidence for Original Antigenic Sin Shaping the Antibody Response to Influenza Vaccination. Clin. Immunol..

[52] Andrews S.F., Huang Y., Kaur K., Popova L.I., Ho I.Y., Pauli N.T., Dunand C.J.H., Taylor W.M., Lim S., Huang M. (2015). Immune History Profoundly Affects Broadly Protective B Cell Responses to Influenza. Sci. Transl. Med..

[53] Halstead S.B., Rojanasuphot S., Sangkawibha N. (1983). Original Antigenic Sin in Dengue. Am. J. Trop. Med. Hyg..

[54] Midgley C.M., Bajwa-Joseph M., Vasanawathana S., Limpitikul W., Wills B., Flanagan A., Waiyaiya E., Tran H.B., Cowper A.E., Chotiyarnwon P. (2011). An In-Depth Analysis of Original Antigenic Sin in Dengue Virus Infection. J. Virol..

[55] Goldstein N.I., Fisher P.B., Fisher P.B. (2007). Surface-Epitope Masking (SEM) BT-Cancer Genomics and Proteomics: Methods and Protocols.

[56] Zarnitsyna V.I., Ellebedy A.H., Davis C., Jacob J., Ahmed R., Antia R. (2015). Masking of Antigenic Epitopes by Antibodies Shapes the Humoral Immune Response to Influenza. Philos. Trans. R. Soc. B Biol. Sci..

[57] Bowen J.E., Addetia A., Dang H.V., Stewart C., Brown J.T., Sharkey W.K., Sprouse K.R., Walls A.C., Mazzitelli I.G., Logue J.K. (2022). Omicron Spike Function and Neutralizing Activity Elicited by a Comprehensive Panel of Vaccines. Science.

[58] Röltgen K., Nielsen S.C.A., Silva O., Younes S.F., Zaslavsky M., Costales C., Yang F., Wirz O.F., Solis D., Hoh R.A. (2022). Immune Imprinting, Breadth of Variant Recognition, and Germinal Center Response in Human SARS-CoV-2 Infection and Vaccination. Cell.

[59] Aydillo T., Rombauts A., Stadlbauer D., Aslam S., Abelenda-Alonso G., Escalera A., Amanat F., Jiang K., Krammer F., Carratala J. (2021). Immunological Imprinting of the Antibody Response in COVID-19 Patients. Nat. Commun..

[60] El Sahly H.M., Baden L.R., Essink B., Doblecki-Lewis S., Martin J.M., Anderson E.J., Campbell T.B., Clark J., Jackson L.A., Fichtenbaum C.J. (2021). Efficacy of the MRNA-1273 SARS-CoV-2 Vaccine at Completion of Blinded Phase. N. Engl. J. Med..

[61] Polack F.P., Thomas S.J., Kitchin N., Absalon J., Gurtman A., Lockhart S., Perez J.L., Marc G.P., Moreira E.D., Zerbini C. (2020). Safety and Efficacy of the BNT162b2 MRNA COVID-19 Vaccine. N. Engl. J. Med..

[62] Logunov D.Y., Dolzhikova I.V., Shcheblyakov D.V., Tukhvatulin A.I., Zubkova O.V., Dzharullaeva A.S., Kovyrshina A.V., Lubenets N.L., Grousova D.M., Erokhova A.S. (2021). Safety and Efficacy of an RAd26 and RAd5 Vector-Based Heterologous Prime-Boost COVID-19 Vaccine: An Interim Analysis of a Randomised Controlled Phase 3 Trial in Russia. Lancet.

[63] Ramasamy M.N., Minassian A.M., Ewer K.J., Flaxman A.L., Folegatti P.M., Owens D.R., Voysey M., Aley P.K., Angus B., Babbage G. (2020). Safety and Immunogenicity of ChAdOx1 NCoV-19 Vaccine Administered in a Prime-Boost Regimen in Young and Old Adults (COV002): A Single-Blind, Randomised, Controlled, Phase 2/3 Trial. Lancet.

[64] Sadoff J., Gray G., Vandebosch A., Cárdenas V., Shukarev G., Grinsztejn B., Goepfert P.A., Truyers C., Fennema H., Spiessens B. (2021). Safety and Efficacy of Single-Dose Ad26. COV2. S Vaccine against COVID-19. N. Engl. J. Med..

[65] Xia S., Zhang Y., Wang Y., Wang H., Yang Y., Gao G.F., Tan W., Wu G., Xu M., Lou Z. (2021). Safety and Immunogenicity of an Inactivated SARS-CoV-2 Vaccine, BBIBP-CorV: A Randomised, Double-Blind, Placebo-Controlled, Phase 1/2 Trial. Lancet Infect. Dis..

[66] Zhang Y., Zeng G., Pan H., Li C., Hu Y., Chu K., Han W., Chen Z., Tang R., Yin W. (2021). Safety, Tolerability, and Immunogenicity of an Inactivated SARS-CoV-2 Vaccine in Healthy Adults Aged 18–59 Years: A Randomised, Double-Blind, Placebo-Controlled, Phase 1/2 Clinical Trial. Lancet Infect. Dis..

[67] Al Kaabi N., Zhang Y., Xia S., Yang Y., Al Qahtani M.M., Abdulrazzaq N., Al Nusair M., Hassany M., Jawad J.S., Abdalla J. (2021). Effect of 2 Inactivated SARS-CoV-2 Vaccines on Symptomatic COVID-19 Infection in Adults: A Randomized Clinical Trial. JAMA.

[68] Dunkle L.M., Kotloff K.L., Gay C.L., Áñez G., Adelglass J.M., Barrat Hernández A.Q., Harper W.L., Duncanson D.M., McArthur M.A., Florescu D.F. (2022). Efficacy and Safety of NVX-CoV2373 in Adults in the United States and Mexico. N. Engl. J. Med..

[69] Keech C., Albert G., Cho I., Robertson A., Reed P., Neal S., Plested J.S., Zhu M., Cloney-Clark S., Zhou H. (2020). Phase 1–2 Trial of a SARS-CoV-2 Recombinant Spike Protein Nanoparticle Vaccine. N. Engl. J. Med..

[70] Wu F., Zhao S., Yu B., Chen Y.-M., Wang W., Song Z.-G., Hu Y., Tao Z.-W., Tian J.-H., Pei Y.-Y. (2020). A New Coronavirus Associated with Human Respiratory Disease in China. Nature.

[71] Choi A., Koch M., Wu K., Chu L., Ma L., Hill A., Nunna N., Huang W., Oestreicher J., Colpitts T. (2021). Safety and Immunogenicity of SARS-CoV-2 Variant MRNA Vaccine Boosters in Healthy Adults: An Interim Analysis. Nat. Med..

[72] Khoury D.S., Docken S.S., Subbarao K., Kent S.J., Davenport M.P., Cromer D. (2022). Predicting the Efficacy of Variant-Modified COVID-19 Vaccine Boosters. medRxiv.

[73] Chalkias S., Harper C., Vrbicky K., Walsh S.R., Essink B., Brosz A., McGhee N., Tomassini J.E., Chen X., Chang Y. (2022). A Bivalent Omicron-Containing Booster Vaccine against COVID-19. N. Engl. J. Med..

[74] Collier A.Y., Miller J., Hachmann N.P., McMahan K., Liu J., Bondzie E.A., Gallup L., Rowe M., Schonberg E., Thai S. (2023). Immunogenicity of BA.5 Bivalent MRNA Vaccine Boosters. N. Engl. J. Med..

[75] Wang Q., Bowen A., Valdez R., Gherasim C., Gordon A., Liu L., Ho D.D. (2023). Antibody Response to Omicron BA.4–BA.5 Bivalent Booster. N. Engl. J. Med..

